# A decade of antimicrobial resistance in *Staphylococcus aureus*: A single center experience

**DOI:** 10.1371/journal.pone.0212029

**Published:** 2019-02-12

**Authors:** Claudia P. Vicetti Miguel, Asuncion Mejias, Amy Leber, Pablo J. Sanchez

**Affiliations:** 1 Department of Pediatrics, Division of Pediatric Infectious Diseases, Nationwide Children’s Hospital - The Ohio State University College of Medicine, Columbus, Ohio, United States of America; 2 Center for Vaccines and Immunity, The Research Institute at Nationwide Children’s Hospital - The Ohio State University College of Medicine, Columbus, Ohio, United States of America; 3 Department of Laboratory Medicine, Nationwide Children’s Hospital, Columbus, Ohio, United States of America; 4 Department of Pediatrics, Division of Neonatology, Center for Perinatal Research, The Research Institute at Nationwide Children’s Hospital, Nationwide Children’s Hospital - The Ohio State University College of Medicine, Columbus, Ohio, United States of America; The University of Hong Kong, HONG KONG

## Abstract

**Background:**

The emergence of community-associated methicillin-resistant *Staphylococcus aureus* (CA-MRSA) resulted in the recommended use of clindamycin and trimethoprim-sulfamethoxazole (TMP-SMX) for suspected *S*. *aureus* infections. The objective of this study was to determine the resistance to methicillin, clindamycin, and TMP-SMX in *S*. *aureus* isolates during a 10-year period.

**Methods:**

Retrospective review of the antimicrobial susceptibilities of all *S*. *aureus* isolates in the outpatient and inpatient settings at Nationwide Children’s Hospital from 1/1/2005 to 12/31/2014. Duplicate isolates from the same site and year and those obtained for MRSA surveillance or from patients with cystic fibrosis were excluded.

**Results:**

Of the 57,788 *S*. *aureus* isolates from 2005–2014, 40,795 (71%) were included. In the outpatient setting, methicillin resistance decreased from 54% to 44% (*p*<0.001) while among inpatient isolates, no significant change was observed. From 2009–2014, resistance to clindamycin among outpatient isolates increased from 16% to 17% (*p* = 0.002) but no significant trend was observed among inpatient isolates (18% to 22%). Similarly, TMP-SMX resistance increased in outpatient *S*. *aureus* isolates from 2005–2014 (0.9% to 4%, *p*<0.001) but not among inpatient isolates. Among both inpatient and outpatient isolates, methicillin-susceptible *S*. *aureus* (MSSA) exhibited higher resistance to both clindamycin and TMP-SMX than MRSA. In addition, resistance to methicillin, clindamycin and TMP-SMX varied widely according to the site of specimen collection.

**Conclusion:**

In a decade where >40,000 *S*. *aureus* isolates were identified at a large pediatric hospital, substantial changes in methicillin, clindamycin, and TMP-SMX resistance occurred. These findings highlight the importance of ongoing surveillance of the local antimicrobial resistance in *S*. *aureus* in order to guide empiric antimicrobial therapy.

## Introduction

The emergence of community-associated methicillin-resistant *Staphylococcus aureus* (CA-MRSA) infections in the late 1990s[1–6] resulted in a shift in the recommendations for antimicrobial therapy for staphylococcal infections from penicillinase-resistant β-lactam agents active against methicillin-susceptible *S*. *aureus* (MSSA) to clindamycin, trimethoprim-sulfamethoxazole (TMP-SMX), and tetracyclines.[7–11] Clindamycin and TMP-SMX are preferred to tetracyclines in children less than 8 years of age because of potential toxicity. The subsequent widespread use of clindamycin and TMP-SMX for presumed and proven staphylococcal infections in children has raised concern for development of antimicrobial resistance in both MRSA and MSSA.[12, 13] Therefore, the objective of this study was to determine the proportion of methicillin, clindamycin, and TMP-SMX resistance in all *S*. *aureus* isolates identified in the outpatient and inpatient settings at Nationwide Children’s Hospital, Columbus, Ohio during a 10-year period. In addition, differences in antimicrobial resistance patterns by type of staphylococcal infection were determined. Such information may impact recommendations for empiric therapy of staphylococcal infections in children.

## Methods

The Institutional Review Board of Nationwide Children’s Hospital approved the study with a waiver of informed consent given that the data were analyzed anonymously. This retrospective study evaluated the antimicrobial susceptibilities to methicillin, clindamycin, and TMP-SMX in all *S*. *aureus* isolates that were identified by the Microbiology Laboratory at Nationwide Children’s Hospital (NCH) from January 1, 2005 to December 31, 2014. The NCH Microbiology Laboratory serves an area of over 3,000 square miles that includes the Columbus Metropolitan area as well as adjacent urban and rural counties (a total of 17 of the 88 counties in Ohio). Before 2014, there was no protocol for empiric antimicrobial therapy for possible staphylococcal infection at NCH, the only pediatric tertiary care center in central Ohio. In 2014, the Pediatric Infectious Diseases division recommended the use of clindamycin for suspected and uncomplicated staphylococcal infections in previously healthy children greater than one year of age that were cared for in non-intensive care settings.

Ascertainment of all positive cultures for *S*. *aureus* was performed using the Laboratory Information Management System. All *S*. *aureus* isolates that grew in culture and were identified by the NCH Microbiology Laboratory from all body and tissue sites were included. Duplicate isolates from the same patient that were cultured from the same site and year, isolates from samples obtained for MRSA surveillance, and isolates from patients with cystic fibrosis who were more likely to harbor multidrug resistant bacteria were excluded.

All staphylococcal isolates were assessed as being from an inpatient or outpatient source based on the patient’s location at the time of specimen collection. Information regarding patient location was obtained using the NCH Enterprise Data Warehouse. Isolates from the Emergency Department were classified according to patient disposition. The isolates also were classified by the body site from where they were obtained: blood, musculoskeletal (e.g. bone tissue, subperiosteal fluid, muscle abscess, and synovial tissue or fluid), respiratory tract (e.g. sputum, tracheal aspirate, and bronchoalveolar lavage [BAL], paranasal sinuses, pleural fluid), peritoneal fluid, urine, cerebrospinal fluid (CSF), skin and soft tissue (SST), and “other” sites (e.g. eye swabs, vaginal/cervical swabs, rectal swabs, skin tissue biopsies, ear drainage, lymph node tissue, esophageal brush specimens).

During the entire study period (2005–2014), antimicrobial susceptibility testing of *S*. *aureus* isolates was performed by the NCH Microbiology Laboratory using mostly the automated VITEK-2 system (bioMérieux, Inc., Durham, NC) for determination of the minimum inhibitory concentrations (MIC). On occasion, E-testing (bioMérieux, Inc., Durham, NC) also was performed to confirm or verify results of the VITEK test. MICs of ≥4 μg/mL and ≥4/76 μg/mL defined resistance to clindamycin and TMP-SMX, respectively, as established by the Clinical and Laboratory Standard Institutes (CLSI). Starting in 2009, a D-test for determination of inducible clindamycin resistance was performed on all erythromycin-resistant *S*. *aureus* isolates. Therefore, trends of clindamycin resistance were analyzed only from 2009 to 2014.

### Statistical analysis

Descriptive statistics were reported as frequency distributions or proportions. Proportion of resistant isolates between different sites were compared using chi-square (χ^2^) test and between MRSA and MSSA using Fisher’s exact test. Antimicrobial resistance trends over time were analyzed using the χ^2^ test for trends. A two-sided *P* < 0.05 was considered to be statistically significant. Statistical analyses were conducted using GraphPad Prism, version 7.0 (La Jolla, CA).

## Results

From 2005 to 2014, 57,788 *S*. *aureus* isolates were cultured and identified at the NCH Microbiology Laboratory. Of these 57,788 isolates, 29% (16,993) were excluded secondary to being duplicate isolates from the same site and patient encounter (n = 8,056), isolates identified during the same year (n = 1,155), or isolates from cystic fibrosis patients (n = 7,782). The 40,795 (71%) remaining *S*. *aureus* isolates were included in the analyses, with methicillin resistance identified in 52% (n = 21,390), clindamycin resistance in 17% (n = 6,988), and TMP-SMX resistance in 2.2% (n = 897) of the isolates. Among all MRSA isolates, 14% (2,913/21,390) and 2% (343/21,390) were resistant to clindamycin and TMP-SMX, respectively. Among MSSA isolates, 21% (4,075/19,405) and 3% (554/19,405) were resistant to clindamycin and TMX-SMX, respectively. All of the *S*. *aureus* isolates were susceptible to vancomycin.

### Proportion of *S*. *aureus* resistance by specimen site

Information on specimen site was available on 37,070 (91%) of the 40,795 *S*. *aureus* isolates. The frequency of antimicrobial resistance for isolates according to sites of infection is provided in Table 1. Overall, methicillin resistance was highest among isolates from pleural fluid (68%, 30/44). Isolates associated with SST infections had significantly higher methicillin resistance (56%, 17,795/31,940) than isolates from blood (40%, 283/702; *p*<0.001) and musculoskeletal sites (36%, 36/113; *p*<0.001).

**Table 1:** Antimicrobial resistance rates to methicillin, clindamycin, and TMP-SMX among *S*. *aureus* isolates from NCH (2005–2014) by site of specimen collection.

Site	Antimicrobial Resistance (%)						
	All *S*. *aureus* (n = 37,070)			MSSA (n = 17,291)		MRSA (n = 19,779)	
	Methicillin	Clindamycin	TMP-SMX	Clindamycin	TMP-SMX	Clindamycin	TMP-SMX
SST (n = 31,940)	17,795/31,940 (55.7)	4,368/31,940 (13.7)	646/31,940 (2)	2,723/14,145 (19.3)	453/14,145 (3.2)	1,645/17,795 (9.2)	193/17,795 (1.1)
Respiratory^a^ (n = 2,258)	941/2,258 (41.7)	971/2,258 (43)	97/2,258 (4.3)	346/1,317 (26.3)	29/1,317 (2.2)	625/941 (66.4)	68/941 (7.2)
Urine (n = 1,270)	309/1,270 (24.3)	345/1,270 (27.2)	33/1,270 (2.6)	184/961 (19.1)	14/961 (1.5)	161/309 (52.1)	19/309 (6.1)
Blood (n = 702)	283/702 (40.3)	203/702 (28.9)	21/702 (3)	90/419 (21.5)	6/419 (1.4)	113/283 (39.9)	15/283 (5.3)
Sinus (n = 178)	64/178 (36.0)	98/178 (55.0)	7/178 (3.9)	45/114 (39.5)	2/114 (1.8)	53/64 (82.8)	5/64 (7.8)
MSK^b^ (n = 113)	36/113 (36.0)	27/113 (24.0)	0/113 (0)	22/77 (28.6)	0/77 (0)	5/36 (13.9)	0/36 (0)
CSF (n = 63)	28/63 (44.4)	25/63 (39.7)	1/63 (1.6)	10/35 (28.6)	0/35 (0)	15/28 (53.6)	1/28 (3.6)
Pleural fluid (n = 44)	30/44 (68.2)	3/44 (6.8)	0/44 (0)	1/14 (7.1)	0/14 (0)	2/30 (6.7)	0/30 (0)
Peritoneal fluid (n = 27)	12/27 (44.4)	13/27 (48.1)	0/27 (0)	5/15 (33.3)	0/15 (0)	8/12 (66.7)	0/12 (0)
Other (n = 475)	281/475 (59.2)	97/475 (20.4)	11/475 (2.3)	60/194 (30.9)	7/194 (3.6)	37/281 (13.2)	4/281 (1.4)

Abbreviations: TMP-SMX, trimethoprim-sulfamethoxazole; NCH, Nationwide Children’s Hospital; MSSA, methicillin-susceptible *Staphylococcus aureus*; MRSA, methicillin-resistant *S*. *aureus*; SST, skin and soft tissue; MSK, musculoskeletal; CSF, cerebrospinal fluid.

^a^ Respiratory isolates included those from sputum, tracheal aspirate, and bronchoalveolar lavage specimens.

^b^ Musculoskeletal isolates included those from bone tissue, subperiosteal fluid, muscle abscesses and synovial tissue or fluid specimens.

Clindamycin resistance was highest among staphylococcal isolates cultured from paranasal sinuses (55%; 98/178) and lowest in those from pleural fluid (7%; 3/44; Table 1). Among blood isolates, 21% (90/419) of MSSA isolates were resistant to clindamycin while 40% (113/283) of MRSA were resistant to clindamycin. Clindamycin resistance was higher in MSSA than MRSA isolates associated with musculoskeletal (29% vs. 14%, *p*<0.001) and SST infections (19% vs. 9%, *p*<0.001).

With respect to TMP-SMX, isolates from the respiratory tract and sinuses demonstrated the highest resistance to TMP-SMX (4% for both). There was no TMP-SMX resistant isolates cultured from musculoskeletal, pleural fluid, and peritoneal fluid samples.

### Proportion of resistance by year and patient location (outpatient vs. inpatient)

Of the 40,795 staphylococcal isolates, 39,520 (97%) had patient location information linked to the isolate with 31,760 (80%) being from outpatients and 7,760 (20%) from inpatients. The number of isolates and proportion of antimicrobial resistance per year in each group are provided in Tables 2 and 3.

**Table 2:** Antimicrobial resistance per year among all *S*. *aureus*, MSSA, and MRSA outpatient isolates from NCH (2005–2014).

Antimicrobial	Proportion of Resistant Isolates (%)										
	2005 (n = 2181)^a^	2006 (n = 2681)	2007 (n = 3002)	2008 (n = 3443)	2009 (n = 3308)	2010 (n = 3279)	2011 (n = 3349)	2012 (n = 3392)	2013 (n = 3542)	2014 (n = 3580)	*p*-value^b^
Methicillin	1184/2181 (54.3)	1512/2681 (56.3)	1693/3002 (56.4)	1937/3443 (56.3)	1746/3308 (52.8)	1751/3279 (53.4)	1658/3349 (49.5)	1704/3392(50.2)	1675/3542 (47.3)	1574/3580 (44.0)	<0.001
Clindamycin^c^											
All *S*. *aureus*	-	-	-	-	531/3308 (16.1)	477/3279 (14.5)	559/3349 (16.7)	600/3392 (17.7)	667/3542 (18.8)	620/3580 (17.3)	0.002
MSSA	-	-	-	-	343/1562 (22.0)	285/1528 (18.7)	360/1690 (21.3)	401/1688 (23.8)	445/1867 (23.8)	429/2006 (21.4)	0.18
MRSA	-	-	-	-	188/1746 (10.8)	192/1751 (11.0)	199/1658 (12.0)	199/1704 (11.7)	222/1675 (13.3)	191/1574 (12.1)	0.07
TMP-SMX											
All *S*. *aureus*	19/2181 (0.9)	25/2681 (0.9)	20/3002 (0.7)	23/3443 (0.7)	40/3308 (1.2)	94/3279 (2.9)	101/3349 (3.0)	95/3392 (2.8)	110/3542 (3.1)	149/3580 (4.2)	<0.001
MSSA	11/997^d^ (1.1)	15/1169 (1.3)	12/1309 (0.9)	15/1509 (1.0)	20/1562 (1.3)	69/1528 (4.5)	68/1690 (4.0)	68/1688 (4.0)	83/1867 (4.4)	108/2006 (5.4)	<0.001
MRSA	8/1184^e^ (0.7)	10/1512 (0.7)	8/1693 (0.5)	8/1937 (0.4)	20/1746 (1.1)	25/1751 (1.4)	33/1658 (2.0)	27/1704 (1.6)	27/1675 (1.6)	41/1574 (2.6)	<0.001

Abbreviations: MSSA, methicillin-susceptible *Staphylococcus aureus*; MRSA, methicillin-resistant *S*. *aureus*; NCH, Nationwide Children’s Hospital; TMP-SMX, trimethoprim-sulfamethoxazole

^a^ Total number of outpatient *S*. *aureus* isolates per year

^*b*^ *p*-value determined using the χ^2^ test for trends.

^c^ Resistance to clindamycin were analyzed in the time period that routine D-test was available (2009–2014) to include isolates with both inducible and constitutive resistance.

^d^ Denominator corresponds to total number of outpatient MSSA isolates per year.

^e^ Denominator corresponds to total number of outpatient MRSA isolates per year.

**Table 3:** Antimicrobial resistance per year among all *S*. *aureus*, MSSA, and MRSA inpatient isolates from NCH (2005–2014).

Antimicrobial	Proportion of Resistant Isolates (%)										
	2005 (n = 536)^a^	2006 (n = 611)	2007 (n = 630)	2008 (n = 666)	2009 (n = 840)	2010 (n = 871)	2011 (n = 858)	2012 (n = 935)	2013 (n = 925)	2014 (n = 888)	*p*-value^b^
Methicillin	269/536 (50.2)	361/611 (59.1)	322/630 (51.1)	348/666 (52.3)	496/840 (59.0)	484/871 (55.6)	451/858 (52.6)	499/935 (53.4)	524/925 (56.6)	530/888 (48.4)	0.54
Clindamycin^c^											
All *S*. *aureus*	-	-	-	-	179/840 (21.3)	191/871 (21.9)	154/858 (17.9)	166/935 (17.8)	203/925 (21.9)	185/888 20.8)	0.88
MSSA	-	-	-	-	78/344 (22.7)	85/387 (22.0)	81/407 (19.9)	83/436 (19.0)	96/401 (23.9)	102/458 (22.3)	0.85
MRSA	-	-	-	-	101/496 (20.4)	106/484 (21.9)	73/451 (16.2)	83/499 (16.6)	107/524 (20.4)	83/530 (19.3)	0.15
TMP-SMX											
All *S*. *aureus*	15/536 (2.8)	9/611 (1.5)	14/630 (2.2)	20/666 (3.0)	16/840 (1.9)	18/871 (2.1)	23/858 (2.7)	23/935 (2.5)	28/925 (3.0)	26/888 (2.9)	0.18
MSSA	3/267 ^d^ (1.1)	1/250 (0.4)	2/308 (0.6)	6/318 (1.9)	4/344 (1.2)	8/387 (2.1)	14/407 (3.4)	10/436 (2.3)	10/401 (2.5)	16/458 (3.5)	0.005
MRSA	12/269 ^e^ (4.5)	8/361 (2.2)	12/322 (3.7)	14/348 (4.0)	12/496 (2.4)	10/484 (2.1)	9/451 (2.0)	13/499 (2.6)	18/524 (3.4)	10/530 (2.3)	0.27

Abbreviations: MSSA, methicillin-susceptible *Staphylococcus aureus*; MRSA, methicillin-resistant *S*. *aureus*; NCH, Nationwide Children’s Hospital; TMP-SMX, trimethoprim-sulfamethoxazole

^a^ Total number of inpatient *S*. *aureus* isolates per year

^*b*^ *p*-value determined using the χ^2^ test for trends.

^c^ Resistance to clindamycin were analyzed in the time period that routine D-test was available (2009–2014) to include isolates with both inducible and constitutive resistance.

^d^ Denominator corresponds to total number of inpatient MSSA isolates per year.

^e^ Denominator corresponds to total number of inpatient MRSA isolates per year.

**Fig 1:**
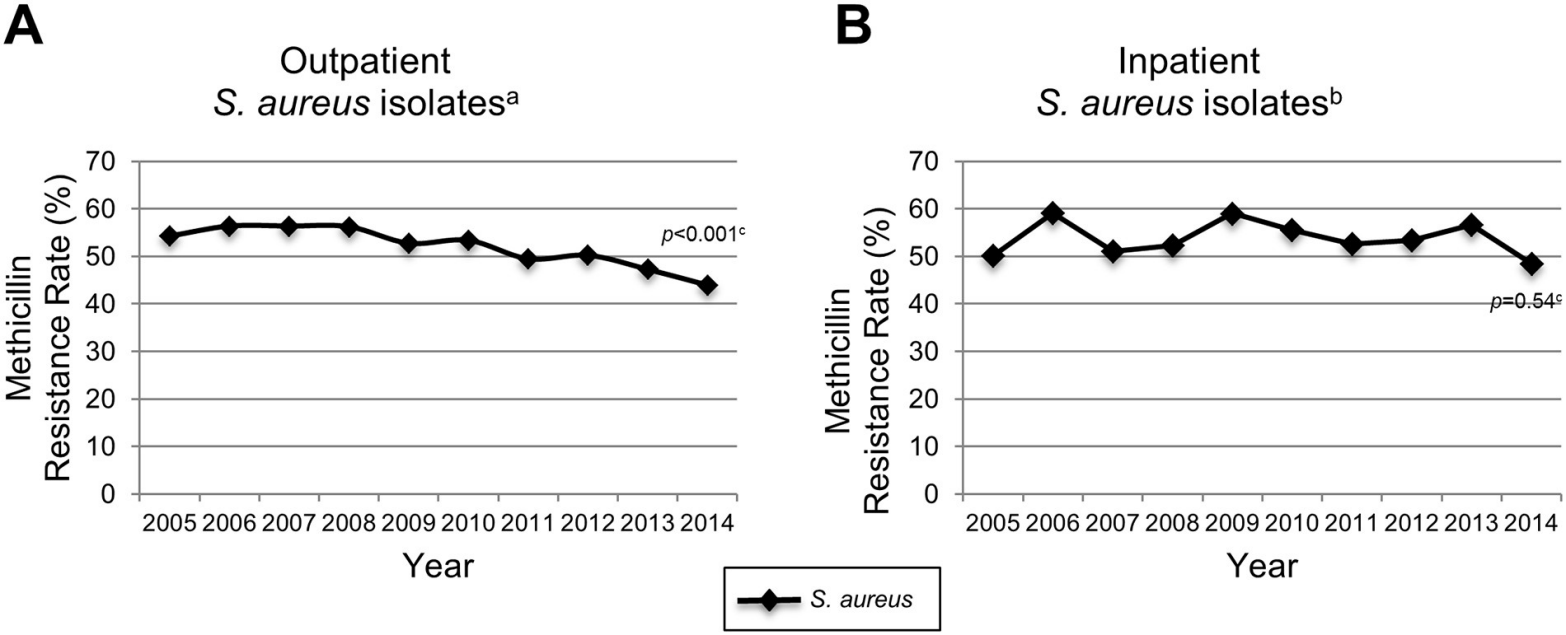
Methicillin resistance by year (2005–2014) in outpatient (A) and inpatient (B) *S*. *aureus* isolates from NCH. NCH indicates Nationwide Children’s Hospital. ^a^Number of outpatient *S*. *aureus* isolates (n = 31,760). ^b^Number of inpatient *S*. *aureus* isolates (n = 7,760). ^c^*p*-value determined using the χ^2^ test for trends.

From 2005 to 2014, the proportion of isolates exhibiting methicillin resistance was 52% (16,435/31,760) and 54% (4,184/7,760) in isolates from outpatients and inpatients, respectively (*p*<0.001). During this time period in the outpatient setting, methicillin resistance decreased from 54% in 2005 to 44% in 2014 (*p*<0.001) while among inpatient isolates, resistance to methicillin varied from 48% to 60% with no significant trends observed (Fig 1).

**Fig 2:**
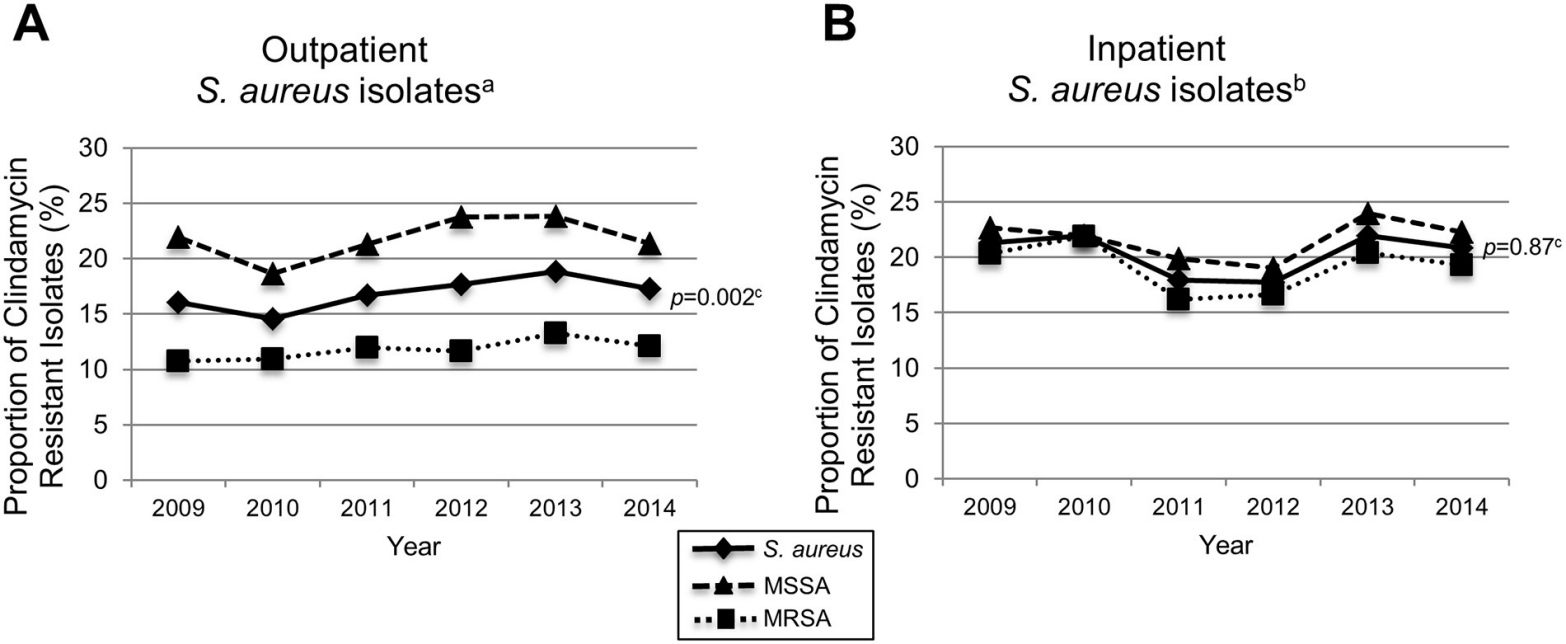
Clindamycin resistance by year (2009–2014) in outpatient (A) and inpatient (B) *S*.*aureus* isolates from NCH. NCH indicates Nationwide Children’s Hospital; MRSA, methicillin-resistant *Staphylococcus aureus*; MSSA, methicillin-susceptible *S*. *aureus*. ^a^Number of outpatient *S*. *aureus* isolates (n = 20,450). ^b^Number of inpatient *S*. *aureus* isolates (n = 5,317). ^c^*p*-value determined using the χ^2^ test for trends.

From 2009 to 2014, clindamycin resistance in all 25,767 *S*. *aureus* isolates was 17% (3,454/20,450) among outpatients and 20% (1,078/5,317) among inpatients (*p*<0.001). In all staphylococcal isolates among outpatients, the resistance to clindamycin increased from 16% in 2009 to 19% and 17% in 2013 and 2014, respectively (Fig 2A, *p* = 0.002). In addition, clindamycin resistance increased significantly in both MSSA and MRSA, and in every year from 2009 to 2014, outpatient MSSA isolates had higher clindamycin resistance than MRSA. Among all inpatient staphylococcal isolates, clindamycin resistance varied from 18% to 22% with no significant trends observed (Fig 2B). During all study years and similar to outpatient isolates, clindamycin resistance in MSSA (22%, 525/2,433) was higher than in MRSA (19%, 553/2,984).

**Fig 3:**
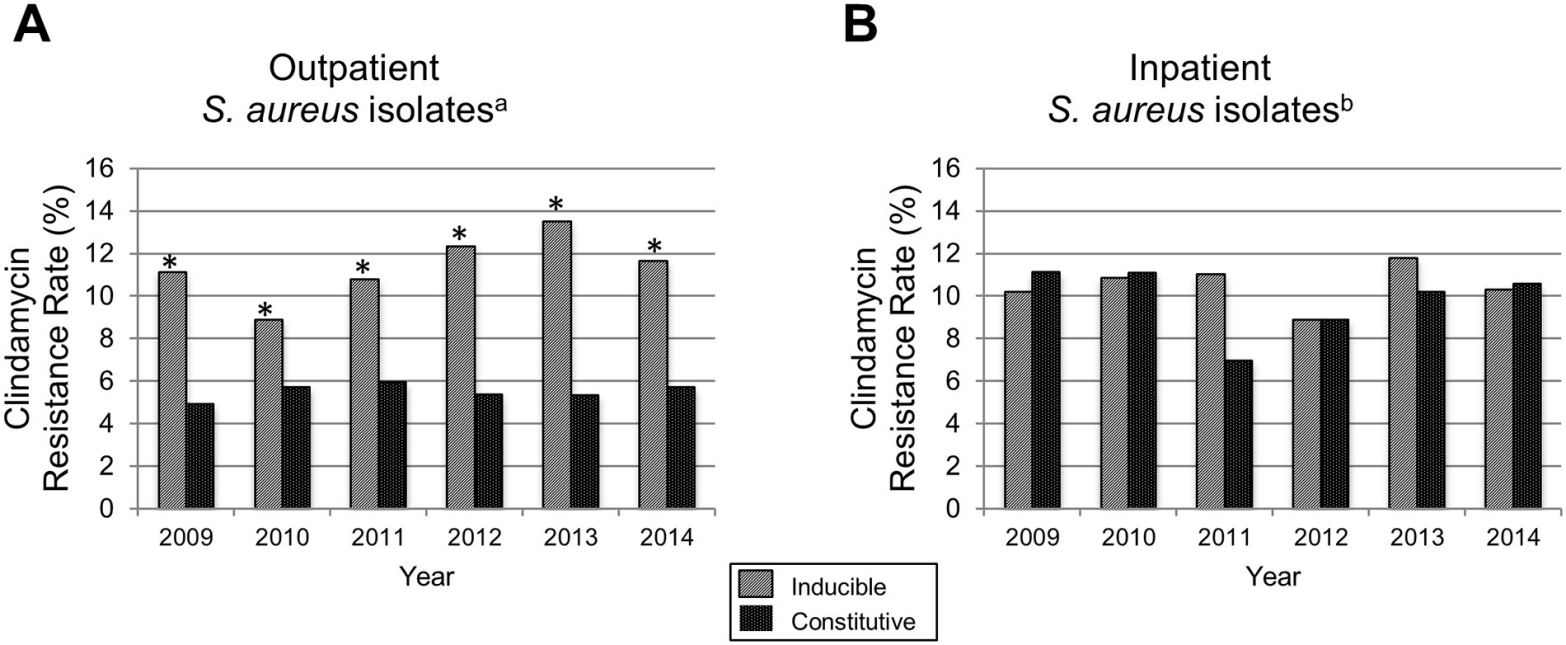
Inducible and constitutive clindamycin resistance by year (2009–2014) in outpatient (A) and inpatient (B) *S*. *aureus* isolates from NCH. NCH indicates Nationwide Children’s Hospital. ^a^Number of outpatient *S*. *aureus* isolates (n = 20,450). ^b^Number of inpatient *S*. *aureus* isolates (n = 5,317). *p<0.05, determined using the χ^2^ test for trends.

Clindamycin resistance in *S*. *aureus* can be constitutive or inducible depending on whether the genes that mediate the most common mechanism of resistance (target site modification by *erm* genes) are always expressed or are induced by the presence of a macrolide antibiotic. In this study, the majority (64%; 2,890/4,532) of clindamycin resistance among all staphylococcal isolates was inducible. Inducible resistance was higher in MSSA than MRSA isolates (86% versus 28%; *p*<0.001). Among all of the clindamycin resistant staphylococcal isolates (n = 4,532), inducible resistance was higher in outpatient (68%, 2,332/3,454) than inpatient (52%, 558/1,078) isolates (*p*<0.001). In addition, among outpatient isolates, inducible but not constitutive clindamycin resistance increased significantly during the study period. However, among inpatient isolates, neither inducible nor constitutive clindamycin resistance changed significantly (Fig 3).

**Fig 4:**
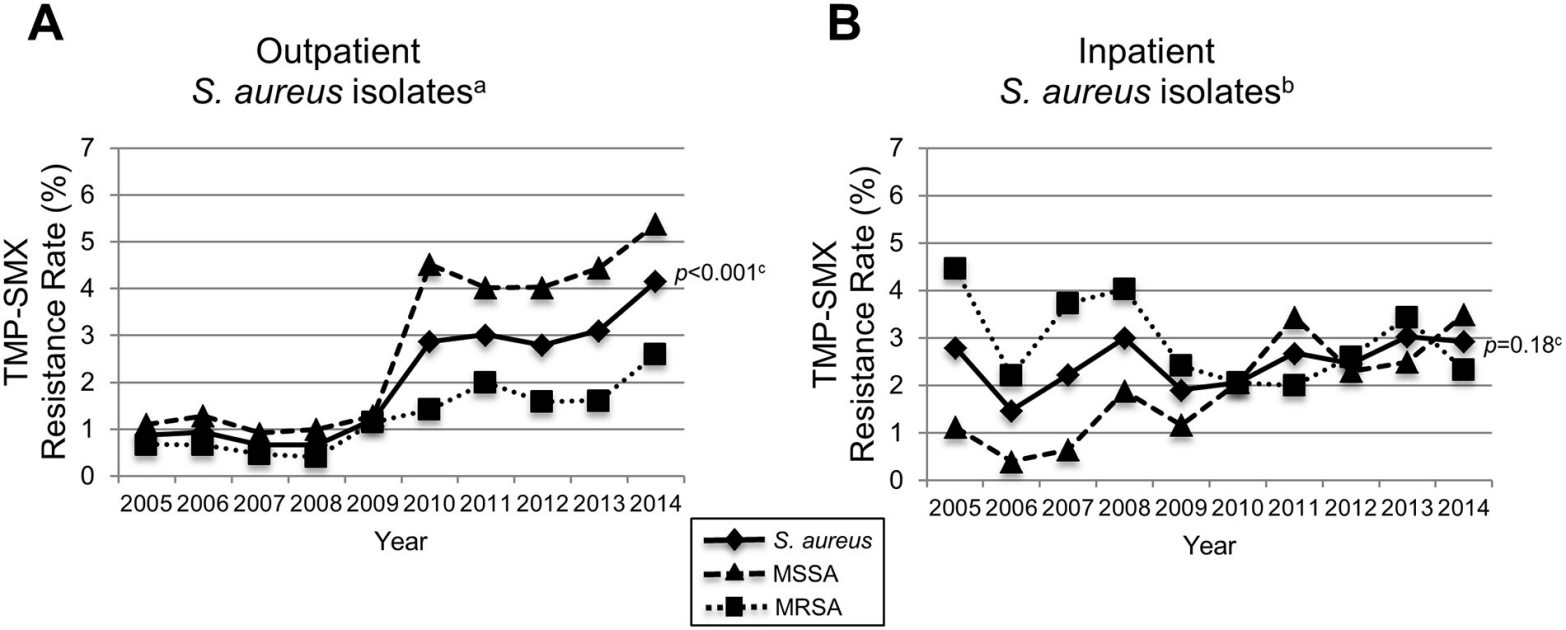
TMP-SMX resistance by year (2005–2014) in outpatient (A) and inpatient (B) *S*.*aureus* isolates from NCH. TMP-SMX indicates trimethoprim-sulfamethoxazole; NCH, Nationwide Children’s Hospital; MRSA, methicillin-resistant *Staphylococcus aureus*; MSSA, methicillin-susceptible *S*. *aureus*. ^a^Number of outpatient *S*. *aureus* isolates (n = 31,760). ^b^Number of inpatient *S*. *aureus* isolates (n = 7,760). ^c^*p*-value determined using the χ^2^ test for trends.

The overall resistance to TMP-SMX from 2005 to 2014 was 2% (676/31,760) in outpatient *S*. *aureus* isolates and 2.5% (192/7,760) among inpatient isolates (*p* = 0.06). Among outpatient isolates, resistance increased in both MSSA and MRSA isolates (Fig 4A, *p*<0.001) with MSSA having higher resistance than MRSA in all years. Among all inpatient staphylococcal isolates, TMP-SMX resistance varied non-significantly from 1% to 3% (Fig 4B). However, among MSSA isolates, TMP-SMX resistance increased from 1% in 2005 to 3.5% in 2014 (*p* = 0.005), while there was no change in resistance among MRSA isolates.

## Discussion

The present study reports resistance to commonly used anti-staphylococcal antimicrobial agents in 40,795 unique *S*. *aureus* isolates from a U.S. pediatric hospital serving a large area in central Ohio. The analysis demonstrated significant differences in antimicrobial resistance by site of specimen collection, higher methicillin and clindamycin resistance among inpatients vs. outpatients, higher clindamycin and TMP-SMX resistance among MSSA vs. MRSA, and importantly, significant changes in antimicrobial resistance patterns during the past decade that included decreasing methicillin resistance but increasing clindamycin and TMP-SMX resistance among outpatient staphylococcal isolates.

SST infections represent the most common pediatric infections in which *S*. *aureus* plays a main role, and among SST isolates, more than 50% were due to MRSA. This proportion of MRSA among SST isolates is substantially higher than the 21% in 2003 and 41% in 2008 that was reported by Karamatsu et al.[14] and the 42% to 20% from 2005 to 2017 reported by Khamash et al.[12] among similar pediatric patients from children’s hospitals in Baltimore and Loma Linda. Other studies that included mainly adult populations found similar methicillin resistance of 50% to 65%.[13, 15, 16] These data support the need for targeting MRSA in the empiric therapy of SST infections. Importantly, clindamycin resistance of 14% among SST isolates found in this study remains within the 10%-15% cut-off recommended by the Infectious Disease Society of America for empiric clindamycin treatment of SST infections.[17] On the other hand, the low (<5%) TMP-SMX resistance in both MRSA and MSSA SST isolates makes TMP-SMX a suitable option.[18]

*S*. *aureus* is the most common bacterial agent that causes invasive infections in children. Among staphylococcal isolates from musculoskeletal sites in this study, MSSA was more frequent than MRSA, and nearly one fourth of the MSSA isolates were resistant to clindamycin. This finding raises important questions about the optimal empiric choice of antistaphylococcal therapy for serious osteoarticular infection in the era of vancomycin stewardship. None of the musculoskeletal staphylococcal isolates were resistant to TMP-SMX, and further study is needed on the use of TMP-SMX for osteoarthritis.[19] Importantly, there was significantly higher clindamycin and TMP-SMX resistance among MSSA than MRSA in both SST and musculoskeletal isolates.

*S*. *aureus* also can cause complicated pneumonias, especially in association with preceding viral infections such as influenza. In this study, pleural fluid isolates had the highest methicillin resistance of almost 70%, but at the same time, the lowest clindamycin resistance (<10%) among both MRSA and MSSA. For this reason, in our institution it appears that clindamycin remains a suitable choice for empiric treatment of staphylococcal pneumonia associated with parapneumonic effusions. None of the pleural fluid isolates were resistant to TMP-SMX. On the other hand, among blood staphylococcal isolates, clindamycin resistance was 29%, with MRSA and MSSA isolates exhibiting resistance rates of 40% and 21%, respectively.

During the 10 year study period, methicillin resistance in *S*. *aureus* significantly decreased, although only among outpatient isolates which is consistent with what has been described previously.[16, 20–25] In a department of defense population consisting of men and women of all ages across the United States, Landrum et al[16] reported a significant decrease in methicillin resistance from 2005 to 2010 in both community-associated SST as well as in community-onset bloodstream infections. Another report focused on SST infections among adult and pediatric patients in northern California, a similar decrease in methicillin resistance in staphylococcal isolates was noted from 2005 to 2009.[23] Sutter el at[24] also described a decrease in methicillin resistance from 2005 to 2014 among pediatric *S*. *aureus* isolates from multiple centers in the U.S., and particularly among isolates from the Midwest starting in 2011. More recently, Khamash et al.[12] noted significant decrease in methicillin resistance from 41% in 2005 to 27% in 2017 among staphylococcal isolates from blood, respiratory, SST, and other anatomic sites in patients younger than 18 years of age. The reason for the decline in methicillin resistance in community-associated infections reported in multiple centers in the U.S. in the last decade is not known, but such information could be of use in the development of strategies to prevent CA-MRSA infections.[26]

Clindamycin has been recommended for empiric therapy of staphylococcal infections. Yet, a significant increase in clindamycin resistance was seen among our outpatient MRSA and MSSA isolates. Increasing proportion of MSSA isolates resistant to clindamycin has been reported previously in a study that analyzed data from 1999 to 2011,[27] and more recently from 2005 to 2017 at the Johns Hopkins Hospital, Baltimore, MD.[12] In addition, Sutter et al[24] also found increasing clindamycin resistance among MSSA isolates from 2005 to 2014 although they were unable to differentiate between constitutive or inducible resistance since not all study sites performed D-testing routinely. In contrast, D-testing has been performed consistently since 2009 at NCH, and an increase in inducible but not constitutive resistance was seen. Given that inducible resistance occurred mostly among MSSA isolates, it is likely that this finding contributed substantially to the overall increase in clindamycin resistance—an observation that has been hypothesized but not reported previously.[24]

*S*. *aureus* has remained highly susceptible to TMP-SMX with most studies reporting resistance rates of <3% in the era of CA-MRSA.[14, 15, 24, 28] More recently, however, Khamash et al.[12] reported that from 2005 to 2017, TMP-SMX resistance in MRSA isolates increased significantly from 2% to 13% and remained relatively stable from 5% to 7% in MSSA isolates. During the ten-year study period of this study, TMP-SMX resistance, although overall low, also increased significantly among outpatient *S*. *aureus* isolates and in both MRSA and MSSA.

The finding of a significant increase in clindamycin and TMP-SMX resistance only among outpatient staphylococcal isolates could be due to frequent use of these antimicrobial agents in the outpatient setting. Although the actual use of clindamycin and TMP-SMX is not known among our patients, these agents are recommended frequentlyfor empiric treatment of staphylococcal infections.[7–9] Therefore, continued surveillance of antimicrobial resistance is of paramount importance in both the outpatient and the inpatient setting.

This study has other limitations, chief of which is the retrospective nature with resultant lack of patient location and specimen collection site in 9% of isolates. In addition, appropriate labeling of clinical sites may not have occurred. Importantly, the inability to separate inpatient staphylococcal isolates into those that were hospital-acquired vs. community-associated was not possible. Identification of specific staphylococcal clones that may have contributed to the observed antimicrobial resistance patterns also was not possible. However, the large number of isolates over a ten-year period and representing a large area of central Ohio adds robust and timely information on antimicrobial resistance.

## Conclusions

Significant changes in antimicrobial resistance patterns were observed among *S*. *aureus* isolates from 2005 to 2014, particularly among isolates obtained in the outpatient setting. These changes included a decrease in methicillin resistance and an increase in clindamycin and TMP-SMX resistance. In addition, MSSA exhibited higher resistance to clindamycin and TMP-SMX than MRSA among outpatient isolates. The increase in clindamycin resistance among outpatient *S*. *aureus* isolates likely was due to an increase in inducible rather than constitutive resistance. Lastly, resistance to methicillin, clindamycin, and TMP-SMX varied widely according to the site of specimen collection. These results highlight the need for ongoing surveillance of local resistance patterns in order to ensure appropriate empiric antimicrobial selection for the treatment of *S*. *aureus* infections.
